# Ultralow detection limit and ultrafast response/recovery of the H_2_ gas sensor based on Pd-doped rGO/ZnO-SnO_2_ from hydrothermal synthesis

**DOI:** 10.1038/s41378-022-00398-8

**Published:** 2022-06-16

**Authors:** Xinxiao Zhang, Jianhai Sun, Kangsong Tang, Hairong Wang, Tingting Chen, Kaisheng Jiang, Tianye Zhou, Hao Quan, Ruihua Guo

**Affiliations:** 1 State Key Laboratory of Transducer Technology, Aerospace Information Research Institute, Chinese Academy of Sciences, 100194 Beijing, China; 2 School of Electronic, Electrical and Communication Engineering, University of Chinese Academy of Sciences, 100049 Beijing, China; 3 School of Mechanical Engineering, Xi’an Jiaotong University, 710049 Xi’an, Shanxi China; 4 Institute of Urban Safety and Environmental Science, Beijing Academy of Science and Technology, 100054 Beijing, China

**Keywords:** Sensors, Nanosensors

## Abstract

Hydrogen (H_2_) sensors are of great significance in hydrogen energy development and hydrogen safety monitoring. However, achieving fast and effective detection of low concentrations of hydrogen is a key problem to be solved in hydrogen sensing. In this work, we combined the excellent gas sensing properties of tin(IV) oxide (SnO_2_) and zinc oxide (ZnO) with the outstanding electrical properties of reduced graphene oxide (rGO) and prepared palladium (Pd)-doped rGO/ZnO-SnO_2_ nanocomposites by a hydrothermal method. The crystal structure, structural morphology, and elemental composition of the material were characterized by FE-SEM, TEM, XRD, XPS, Raman spectroscopy, and N_2_ adsorption–desorption. The results showed that the Pd-doped ZnO-SnO_2_ composites were successfully synthesized and uniformly coated on the surface of the rGO. The hydrogen gas sensing performance of the sensor prepared in this work was investigated, and the results showed that, compared with the pure Pd-doped ZnO-SnO_2_ sensor, the Pd-doped rGO/ZnO-SnO_2_ sensor modified with 3 wt% rGO had better hydrogen (H_2_)-sensing response of 9.4–100 ppm H_2_ at 380 °C. In addition, this sensor had extremely low time parameters (the response time and recovery time for 100 ppm H_2_ at 380 °C were 4 s and 8 s, respectively) and an extremely low detection limit (50 ppb). Moreover, the sensor exhibited outstanding repeatability and restoration. According to the analysis of the sensing mechanism of this nanocomposite, the enhanced sensing performance of the Pd-doped rGO/ZnO-SnO_2_ sensor is mainly due to the heterostructure of rGO, ZnO, and SnO_2_, the excellent electrical and physical properties of rGO and the synergy between rGO and Pd.

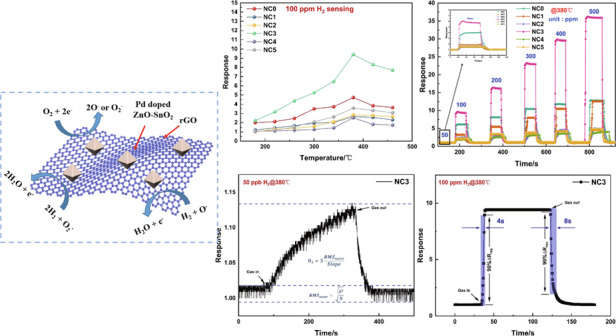

## Introduction

As a new type of green energy, hydrogen (H_2_) can help achieve the global strategic goal of reducing carbon emissions^1^. Hydrogen is considered to be one of the most promising energy sources in the future due to its abundant reserves, environmental friendliness, and high energy density. As a new fuel carrier, hydrogen has been widely used in the aerospace, petrochemical, and biomedical industries. However, due to its wide flammable range (4–75%), low ignition energy (0.002 mJ), and fast burning rate, the accidental leakage of hydrogen during production, use, and transportation is an important safety issue^2,3^. To maintain a stable hydrogen ecosystem, every part of the system, including the source, infrastructure, and fuel storage system, must be reliable. Therefore, the development of hydrogen sensors with high sensitivity, low detection limit, and fast response is of great significance for human safety and environmental protection^4^.

Tin(IV) oxide (SnO_2_) is a typical n-type semiconductor with a wide band gap (Eg = 3.6 eV) and is a very popular gas-sensitive material due to its high conductivity, good stability, and ease of synthesis^5^. It is used to detect various gases. In principle, gas detection mainly depends on the electron transfer on the surface of the semiconductor gas-sensing material, which is usually divided into electron capture and electron release^6^. This is also the major reason why the gas-sensing material experiences a change in resistance under the target gas atmosphere. However, pure SnO_2_ usually suffers from a long response recovery time, high detection limit, and poor sensitivity. Common methods of improving the gas sensing performance of SnO_2_ include the formation of nano-heterostructures^7–9^ and the doping of metal nanoparticles^10–12^. The addition of other metal oxides to form nano-heterostructures and the resistance change is an effective strategy because they modulate the electron depletion layer. ZnO is another n-type semiconductor with a wide band gap (Eg = 3.37 eV)^13^. The nano-heterostructures formed by the combination of ZnO and SnO_2_ are expected to improve the gas sensing performance^14,15^. Zhang et al. synthesized a hierarchical SnO_2_/ZnO nanostructure by a two-step hydrothermal method and realized ppb-level detection of NO_2_, and the response and recovery times were both <60 s^7^. Yang et al. used a one-step hydrothermal method to prepare a novel heterostructured nanocomposite composed of a ZnO backbone and SnO_2_ branches, which exhibited a fast response to ethanol (18.1 s–100 ppm)^16^. Li et al. synthesized an ethanol sensor based on a ZnO-SnO_2_ heterojunction. They synthesized SnO_2_ nanofibers by electrospinning, and a ZnO shell was grown on the nanofibers by a hydrothermal method. The response of the material was 11 times that of the SnO_2_ sensor^8^. ZnO-SnO_2_ heteromaterials with different structures have better gas sensing properties than pure SnO_2_ materials.

In recent years, graphene has received extensive attention in many fields, including gas sensing applications, due to its high electrical conductivity, large specific surface area, and excellent chemical stability^17^. However, pristine graphene is less sensitive to gases. Several recent studies have shown that reduced graphene oxide (rGO) can be formed by oxidizing and reducing graphene^18,19^. Compared with graphene, while maintaining the excellent properties of high electrical conductivity and high specific surface area, rGO has more abundant surface functional groups, higher catalytic activity, and more active sites, which are conducive to the adsorption of gases^20^. The main effects of rGO on gas sensing materials are as follows: (1) it forms a p-n heterojunction with an n-type metal oxide semiconductor, which increases the resistance of the material and increases the resistance change; (2) it has good conductivity and can detect small changes in electrical resistance caused by gas adsorption; and (3) it has a high specific surface area and many defect sites, which improve gas adsorption. Drmosh et al. used a method combining liquid-phase pulsed laser ablation and direct current sputtering to prepare a ternary rGO/ZnO/Pt material for low-concentration hydrogen detection, and the response was 10 times that of a pure ZnO material^21^. Achary et al. synthesized a ZnFe_2_O_4_-Pd-decorated rGO nanocomposite by a microwave-assisted method, whose response to 200 ppm hydrogen was 11.43%, and the response time and recovery time were 18 and 39 s, respectively^22^. Sivakumar et al. used a solution-based hydrothermal method to fabricate a SnO_2_/rGO hybrid sensor with a high response (88.9), fast response (12 s), and recovery time (34 s) to NO_2_^23^.

Palladium (Pd) has been used as an effective catalyst for hydrogen detection due to its affinity for hydrogen^24^. In a hydrogen atmosphere, Pd destroys the bond between the hydrogen atoms to generate PdH_x_, resulting in lattice expansion and resistance change^25^. In addition, the “spillover effect” of Pd can effectively enhance the response of gas-sensing materials and improve the selectivity and sensitivity to hydrogen^26^. To date, Pd-doped nanometal oxide-based hydrogen sensors prepared by a variety of different methods have been reported. Zhang et al. used a hydrothermal method to prepare a Pd-modified SnO_2_/MoS_2_ hydrogen sensor that had good sensitivity and a fast response time for 30–5000 ppm hydrogen^27^. Wu et al. prepared a mesoporous Pd-loaded WO_3_ hydrogen sensor by a hard template method; this sensor could detect hydrogen at room temperature^28^. Duan et al. used a template-free hydrothermal method to synthesize different amounts of Pd-modified SnO_2_ spherical composites as hydrogen sensing materials. Under 10 mW/cm^2^ UV light irradiation, the response to 200 ppm hydrogen was 18.1, and the response time and recovery time were 2.2 and 17.4 s, respectively^26^.

In this paper, palladium-doped rGO/ZnO-SnO_2_ quaternary nanocomposites were prepared by a hydrothermal method for use as gas sensing materials in hydrogen sensors. Their morphology, structure, elemental composition, and lattice parameters were analyzed by various characterization methods, which proved the successful preparation of the material. Additionally, the hydrogen-sensing properties of materials with different modification amounts of rGO were tested under different conditions, and the optimal modification amount of rGO was obtained. The sensing mechanism of the quaternary nanocomposites is also discussed.

## Experiment

### Synthesis of ZnO

All reagents used were of analytical grade, purchased from Sinopharm Group, and used without further purification. ZnO nanorods were prepared by a one-step hydrothermal method. In a typical synthesis process, 4.4 g of zinc acetate dihydrate (Zn(Ac)_2_·2H_2_O) was dissolved in deionized water and magnetically stirred for 30 min to form a uniform mixture, and 3.16 g of ammonium bicarbonate (NH_4_HCO_3_) was added to it to form a mixed solution. A white precipitate formed, the mixture was magnetically stirred again for 30 min, and then it was transferred to a 100 mL Teflon-lined stainless steel autoclave. The autoclave was placed in an oven at 120 °C for 3 h and subsequently cooled to room temperature naturally. The reactants were collected by centrifugation and washed several times with ethanol and deionized water, and the washed reactants were placed in a 60 °C oven to dry overnight. After drying, they were calcined in a muffle furnace at 500 °C for 2 h to yield ZnO nanorods.

### Preparation of graphene oxide

Graphene oxide was prepared using a modified Hummer method^29^. In short, 180 mL of concentrated sulfuric acid, 8 g of graphite powder, and 4 g of sodium nitrate were first mixed and then stirred in an ice bath for 30 min. Then, 14 g of potassium permanganate was added while stirring, 80 mL of distilled water was added, and 30 mL of 5% hydrogen peroxide was added. Finally, the obtained mixed solution was centrifuged repeatedly, washed three times with hydrochloric acid solution and deionized water each, and then dried at 60 °C to yield graphene oxide.

### Ultrasonic dispersion of graphene oxide

Aqueous dispersions of GO with different weight ratios were prepared. In this method, GO powder and L-ascorbic acid were mixed and dissolved in deionized water at a mass ratio of 1:10 and ultrasonically dispersed for 1 h to form a uniform brown dissociated solution. The weight ratios of the GO and ZnO-SnO_2_ gas-sensing nanocomposites were 0, 1, 2, 3, 4, and 5 wt%, abbreviated as NCO, NC1, NC2, NC3, NC4, and NC5, respectively.

### Synthesis of Pd-doped rGO/ZnO-SnO2 nanocomposites

**Fig. 1:**
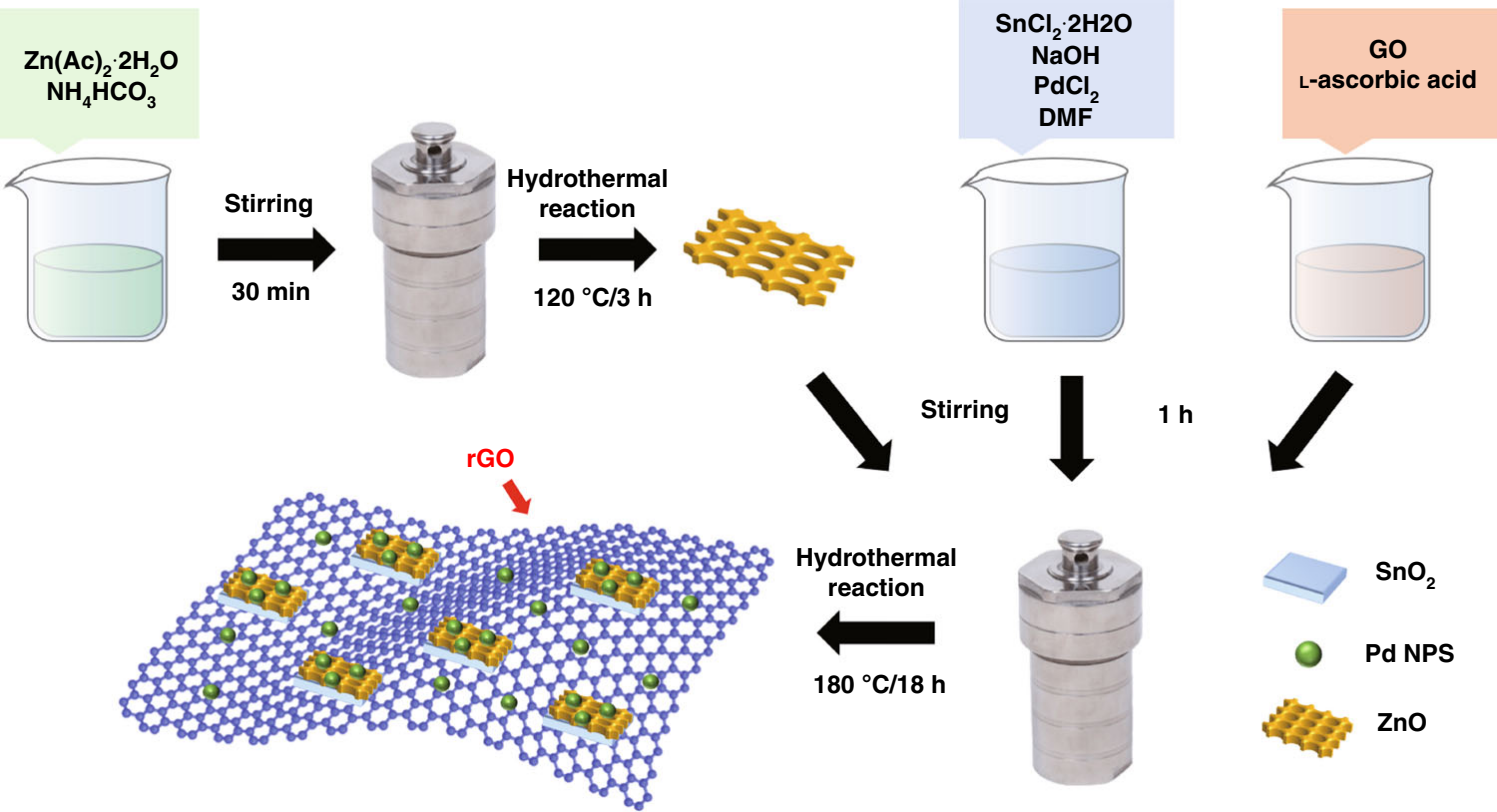
**Experimental set-up for the nanocomposites**. The fabrication process of the Pd-doped rGO/ZnO-SnO_2_ nanocomposites.

The nanocomposites were prepared by a hydrothermal method. First, 1.13 g of stannous chloride dihydrate (SnCl_2_·2H_2_O) was dissolved in deionized water and magnetically stirred for 15 min to form a uniform milky-white suspension, and 12 mL of *N*,*N*-dimethylformamide (DMF) and 0.4 g of NaOH were added. A pale yellow precipitate formed. After stirring for 20 min, 0.135 g of ZnO nanorods prepared in step 1 and 0.059 g of palladium(II) chloride (PdCl_2_) were added, and the mixture was stirred again for 30 min to form a mixed solution. The mixed solution was added to GO dissociation solutions of different weight ratios under stirring conditions, and after magnetic stirring for 1 h, they were transferred to a 100 mL Teflon-lined stainless steel autoclave, sealed, and reacted at 180 °C for 18 h, and cooled naturally. The reactants were collected by centrifugation and washed several times with ethanol and deionized water, and the washed reactants were placed in an oven at 60 °C overnight drying. After drying, they were placed in a muffle furnace for calcination at 500 °C for 2 h to finally obtain Pd-doped rGO/ZnO-SnO_2_ nanocomposites. The experimental set-up for the nanocomposite is shown in Fig. 1.

### Material characterization

The crystal phases of the prepared products were analyzed by X-ray diffraction (XRD, D8-Advance, Bruker) using Cu-Kα radiation (*λ* = 1.54060 Å) in the range of 10–90°. The surface morphology of the prepared composites was characterized by field emission scanning electron microscopy (FE-SEM, SU8020, Hitachi). The detailed structure and composition characterization of the samples were observed with a transmission electron microscope (TEM, Talos F200X, FEI) equipped with an energy-dispersive X-ray (EDX) spectrometer at an accelerating voltage of 200 kV. The Raman spectrum of the prepared sample was obtained by Raman spectroscopy (Raman, inVia Qontor, Renishaw) with an excitation wavelength of 532 nm. The composition and oxidation state of the products were explored by X-ray photoelectron spectroscopy (XPS, 250Xi, Thermo Scientific) with a microfocus X-ray monochromatic source. The specific surface area of the samples was determined by the N_2_ adsorption-desorption isotherms using the Brunauer–Emmett–Teller (BET) method with a physisorption analyzer (TriStar II 3020, Micromeritics).

### Gas-sensor fabrication and response test

The hydrogen sensor was fabricated using the prepared nanocomposite powder. The calcined nanocomposites were ground and crushed using an agate mortar. A mixed solution of ethylene glycol:deionized water in a 1:1 ratio was used to make a paste slurry, the slurry was applied to the surface substrate of the sensor chip, and then the electrodes of the sensor chip were connected to the external tube shell by pressure welding technology to complete the hydrogen sensor. These hydrogen sensors were installed in homemade sensor modules for testing. The resistance “*R*_a_” of the sensor in the air and the resistance “*R*_g_” in the presence of the test gas were obtained. Finally, the following formula was used to calculate the sensor response (*S*)^30^:

1 $$S = R_{\rm{a}}/R_{\rm{g}}$$

The response time and recovery time were defined as the time it took for the sensor to reach a stable response or to recover to 90% of the stable baseline.

## Results and discussion

### Material characterization

**Fig. 2:**
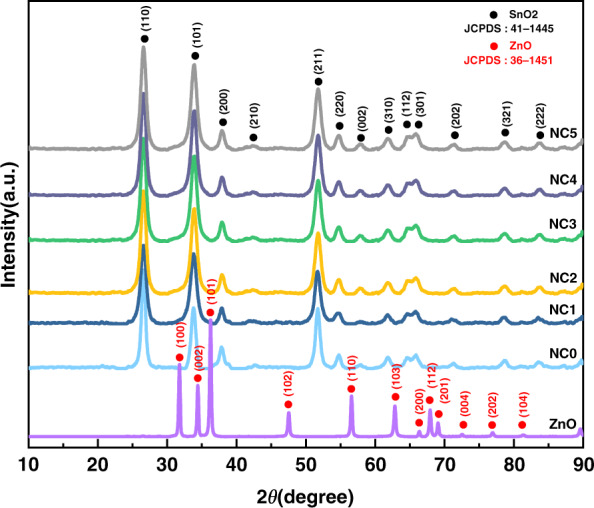
**XRD characterization of nanocomposites.** XRD patterns of ZnO, NC0, NC1, NC2, NC3, NC4, and NC5.

The crystal phases of the prepared products were analyzed by X-ray diffraction (XRD, D8-Advance, Bruker) using Cu-Kα radiation (*λ* = 1.54060 Å) in the range of 10–90°. The XRD patterns of the prepared ZnO and all samples are shown in Fig. 2. In the XRD pattern, there are only single diffraction peaks corresponding to ZnO and SnO_2_, and no redundant diffraction peaks are present, which proves the high purity of the sample. In the ZnO XRD pattern, the peaks at 31.77°, 34.42°, 36.25°, 47.54°, 56.60°, 62.86°, 66.38°, 67.96°, 69.10°, 72.56°, 76.95°, and 81.37° correspond to the (100), (002), (101), (102), (110), (103), (200), (112), (201), (004), (202) and (104) planes, labeled hexagonal ZnO (JCPDS36–1451). In the XRD pattern of the samples, the diffraction peaks at 26.61°, 33.89°, 37.95°, 42.63°, 51.78°, 54.76°, 57.82°, 61.87°, 64.72°, 65.94°, 71.28°, 78.71°, and 83.71° corresponding to the SnO_2_ planes (110), (101), (200), (210), (211), (220), (002), (310), (112), (301), (202), (321), and (222) are indexed as tetragonal rutile pure phase SnO_2_ (JCPDS41–1445). In the XRD patterns, the diffraction peaks of ZnO and SnO_2_ are obvious, proving the successful synthesis of the composites. Due to the large proportion of SnO_2_ in the sample, the XRD pattern of ZnO is poor, and the subsequent characterization further illustrates the successful synthesis of ZnO-SnO_2_. To confirm the presence of rGO, the Raman spectra of GO and all samples were obtained.

**Fig. 3:**
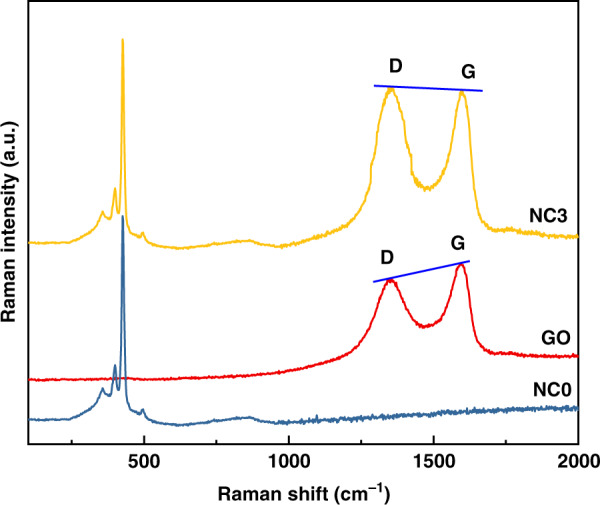
**Raman spectroscopy characterization of nanocomposites.** Raman spectra of GO, NC0, NC1, NC2, NC3, NC4, and NC5.

Figure 3 shows the Raman spectra of GO, pure Pd-doped ZnO-SnO_2_, and Pd-doped rGO/ZnO-SnO_2_ ternary composites. The figure shows that the Raman spectrum of GO has two strong peaks, the D peak and the G peak, at wavenumbers of 1351 cm^−1^ and 1589 cm^−1^, respectively^31,32^. The D peak is attributed to atomic disorder and the surface defects of graphene, while the G peak is attributed to the first-order scattering of the E_2g_ phonons of the sp^2^ carbon atoms^33^. In the Raman spectra of the rGO/ZnO-SnO_2_ ternary composite, when GO was doped into the nanocomposite and reduced to rGO, the relative intensity ratio (*I*_D_/*I*_G_) of the D peak to the G peak changed. The *I*_D_/*I*_G_ value in the composite (*I*_D_/*I*_G_ = 1.01) is larger than that of GO (*I*_D_/*I*_G_ = 0.91). This result suggests that there are more local sp^3^ defects and increased disorder in the sp^2^ carbon network after GO reduction. It is demonstrated that GO was completely reduced to rGO in the ternary composite, and rGO contained many active sites. In addition, the Raman spectrum of the rGO/ZnO-SnO_2_ ternary composite contained the characteristic peaks of the pure ZnO-SnO_2_ Raman spectrum^23,33^. The 390 cm^−1^ and 431 cm^−1^ peaks correspond to the A_1_(TO) and E_2_(h) vibrational photon modes of ZnO, respectively. The peak located at 667 cm^−1^ is assigned to the fundamental A_2g_ mode, characteristic of the tetragonal phase of SnO_2_. The results indicate that a stable heterostructure forms between rGO and ZnO-SnO_2_, which endows the samples with potentially excellent gas-sensing properties.

**Fig. 4:**
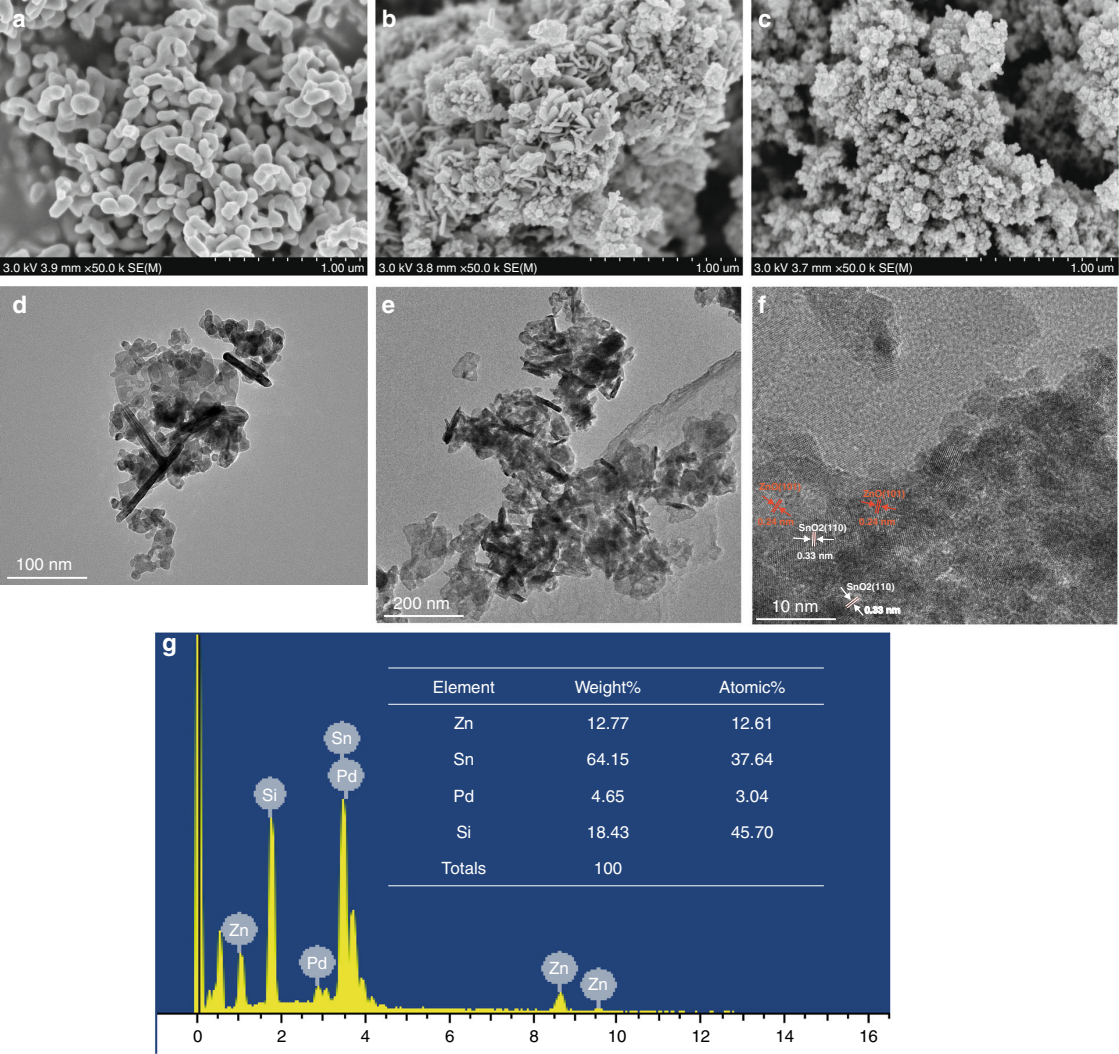
**SEM and TEM characterization of nanocomposites.** SEM image of **a** ZnO, **b** NC0, **c** NC3; TEM image of **d** NC0, **e**, **f** NC3; **g** EDX of NC3.

The morphologies of pure ZnO, NCO, and NC3 samples were observed using field emission scanning electron microscopy (FE-SEM). Figure 4a shows the FE-SEM image of ZnO. ZnO has a nanorod structure with a length of approximately 200–300 nm assembled together to form a branch-like structure. The binary nanocomposites formed by the self-assembly of ZnO nanorods and SnO_2_ nanosheets of different sizes are shown in Fig. 4b, an n-n-type heterojunction is formed between ZnO nanorods and SnO_2_ nanosheets. As shown in Fig. 4c, after the material is modified with rGO, the size of the material decreases, and the material becomes more uniform. rGO is uniformly coated on the surface of the ZnO-SnO2 material. Figure 4d shows the transmission electron microscopy (TEM) image of the NCO sample, where the ZnO nanorods and SnO2 nanosheets can be clearly seen, which again confirms the FE-SEM results. As shown in Fig. 4e, f, 2D-structured rGO was observed in the NC3 sample. In the composite lattice fringes, the interplanar spacings are 0.24 nm and 0.33 nm, corresponding to the ZnO and (101) plane and SnO_2_ (110) plane, respectively. To further determine the presence of elements in the NC3 sample, EDX analysis was performed, and the results are shown in Fig. 4g. The corresponding peaks of Zn, Sn, and Pd can be clearly seen in the spectrum, and the amount is basically the same as that added during the preparation process. The above results demonstrate the successful synthesis of ZnO-SnO_2_ heterostructures and the efficient doping and modification of Pd nanoparticles and rGO.

**Fig. 5:**
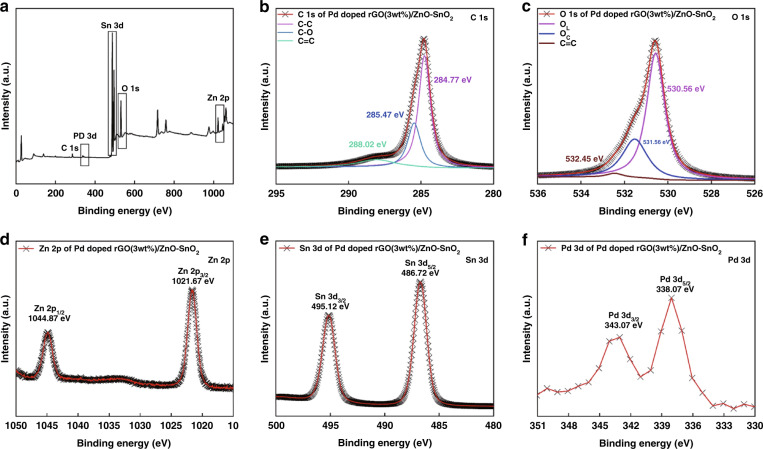
**XPS spectra of the NC3 composite.** **a** full spectrum; **b** C 1s; **c** O 1s; **d** Zn 2p; **e** Sn 3d; **f** Pd 3d.

The surface element composition of NC3 samples and their corresponding chemical binding states were investigated by XPS (Fig. 5). Figure 5a is the full spectrum of the sample. The presence of C, O, Zn, Sn, and Pd can be clearly observed; in the spectrum, the peak characteristic of C is small and there is no impurity peak. The energy level maps of the C 1s, O 1s, Zn 2p, Sn 3d, and Pd 3d core levels are shown in Fig. 5b–f. In Fig. 5b, the C 1s spectrum can be reversed into three distinct peaks at 288.02, 285.47, and 284.77 eV, which correspond to C-C, C-O, and C=C in the aromatic ring, respectively. The O 1s spectrum is shown in Fig. 5c; this spectrum can be divided into three peaks at different positions. The main peak at 530.56 eV is assigned to oxygen ions (O_L_) in the crystal lattice, while the main peak at 531.56 eV is assigned to absorbed oxygen ions (O_C_). In general, O_L_ is caused by oxygen ions in the lattice, which is considered to be fairly stable and does not contribute to the gas response^34^. Additionally, oxygen ions are adsorbed by the material to form O_C_, and these oxygen ions play an important role in the gas sensing performance, which will be further discussed in the gas sensing mechanism section. In addition, the peak corresponding to the C=O occurs at 532.45. In Fig. 5d, the Zn 2p peak represents the Zn 2p_3/2_ and 2p_1/2_ orbitals in doublet states with binding energies of 1021.67 and 1044.87 eV, respectively. In Fig. 5e, the two peaks at 486.72 and 495.12 eV represent the binding energies of the Sn 3d_5/2_ and 3d_3/2_ orbitals. The above results prove that Zn is present in the +2 valence state in the gas sensing material, while Sn is present in the +4 valence state. Finally, the two peaks at 338.07 and 343.07 eV in Fig. 5f represent the binding energies of the Pd 3d_5/2_ and 3d_3/2_ orbitals, respectively, indicating the successful synthesis of the ZnO-SnO_2_ matrix and the efficient doping of Pd nanoparticles.

Table 1 shows the BET specific surface area of the prepared NCO and NC3 samples. The specific surface areas of NC0 and NC3 are 31.3071 and 60.9185 m^2^/g, respectively. After the modification of rGO, the 3 wt% rGO/ZnO-SnO_2_ composite has a larger specific surface area than the pure ZnO-SnO_2_ composite due to the high specific surface area of rGO and the effective modification of rGO. The higher specific surface can provide more active sites, which is beneficial to the surface reaction in the gas sensing process, resulting in higher response and faster response/recovery capability of the gas sensor^35^.

**Table 1:** BET-specific surface area of NC0 and NC3 samples

Samples	Specific surface area (m^2^/g)
NC0	31.3071
NC3	60.9185

### Sensing properties for H_2_

**Fig. 6:**
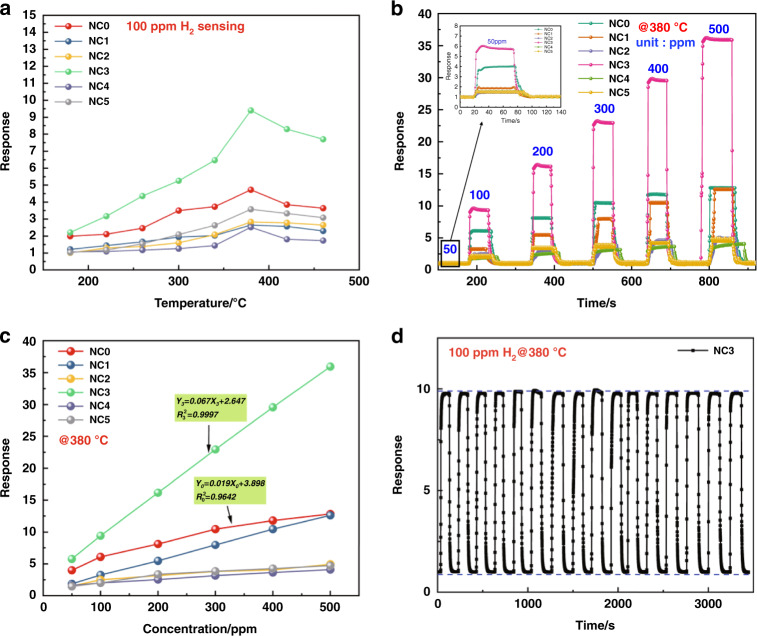
Gas sensing properties of the Pd-doped rGO/ZnO-SnO_2_ based sensors. **a** Response of all samples at different temperatures to 100 ppm H_2_; **b** dynamic response curves of all samples to different concentrations of hydrogen at 380 °C; **c** transient response curves of all samples to different concentrations of hydrogen at 380 °C; **d** repeatability of NC3 to 100 ppm H_2_ at 380 °C.

It is well known that temperature is one of the key parameters of a sensor. The working temperature mainly affects the adsorption and desorption process and reaction kinetics of the gas on the surface of the gas-sensing material, which plays a key role in the response. Therefore, we first analyzed the response temperature function of the sensor. The test results of the Pd-doped rGO/ZnO-SnO_2_ sensor are shown in Fig. 6a. The results show that the six samples all exhibited a trend of increasing first and then decreasing in response to 100 ppm H_2_ at different temperatures. This trend occurred because the increase in temperature can satisfy the activation energy required for chemical adsorption and increase the amount of gas adsorption. The increase in temperature can also provide energy to reduce the reaction barrier of oxygen species and make the redox reaction more efficient. However, when the temperature exceeds the optimum temperature, the adsorbed hydrogen molecules begin to desorb, and the sensor response decreases. The curve in Fig. 6a shows that the NC3 material has a maximum hydrogen response of 9.4 at 380 °C, which is 2 and 2.7 times that of NC0 and NC5, respectively. Compared with pure ZnO-SnO_2_ composites, the materials doped with 3 wt% rGO exhibit a better hydrogen response.

Figure 6b shows the dynamic responses of all sample sensors to 50–500 ppm H2 at an operating temperature of 380 °C. The gas responses of the six sensors improve with increasing hydrogen concentration and are able to recover to the initial state. It has been demonstrated that the sensor can be used for hydrogen detection over a wide concentration range and has good recovery performance. Clearly, the gas response of the NC3 sensor is higher than that of the sensors made from the other five samples at each hydrogen concentration.

To investigate the sensitivity of the proposed hydrogen sensor at the optimum operating temperature, the transient response of the proposed sensor at hydrogen concentrations of 50–500 ppm was investigated, as shown in Fig. 6c. The results show that the sensor response has a good linear relationship with the hydrogen concentration, and NC3 is more sensitive than the other sensors, with a linear correlation of 0.9997, which confirms that the sensor based on the NC3 sample material can be used for hydrogen detection.

To meet the needs of practical applications, the repeatability of the NC3 sensor was tested. Figure 6d shows the repeatability curve of the NC3 sensor exposed to 100 ppm hydrogen at 380 °C. The sensor has been tested for a total of 16 cycles, and the response values are almost the same each time, with no obvious change, and can return to the initial state after the response, which proves that the sensor has good repeatability and recovery. The high stability and high repeatability of the sensor can be attributed to the stability of the nanocomposite structure.

**Fig. 7:**
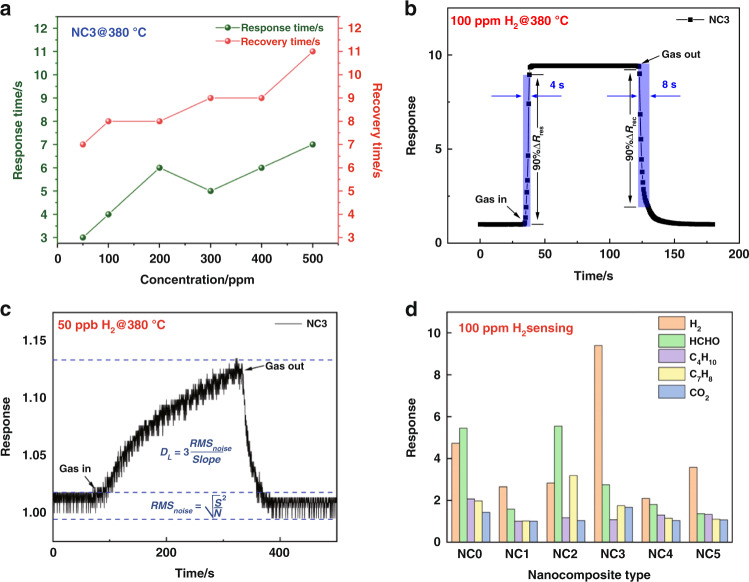
Gas sensing properties of the NC3 sensor. **a** Response time and recovery time curves of the NC3 sensor to different hydrogen concentrations at 380 °C; **b** response transients of the NC3 sensor to 100 ppm H_2_ at 380 °C; **c** response transients of the NC3 sensor to 50 ppb H_2_ at 380 °C; **d** the selectivity of the NC3 sensor to 100 ppm concentrations of different gases at 380 °C.

The response time (*T*_*RES*_) and recovery time (*T*_*REC*_) are defined as the time for the sensor to reach 90% of its final response and recovery, respectively. It is a key parameter in a gas sensor, and a fast and effective response to the target gas is a must-have feature of an excellent sensor. As shown in Fig. 7a, the response time and recovery time of the NC3 sensor at the optimum operating temperature to different H_2_ concentrations were investigated. Within the range of 50-500 ppm H_2_, the response time and recovery time are both less than 10 s. The result that the response time is shorter than the recovery time shows that the adsorption and reduction reactions of H_2_ gas on the material surface are usually faster than the desorption and oxidation reactions on the material surface. The transient response of the NC3 sensor to 100 ppm H_2_ at 380 °C is shown in Fig. 7b. The response time and recovery time of the NC3 sensor to 100 ppm H_2_ at 380 °C are 4 s and 8 s, respectively. The sensor exhibits an outstanding fast response performance. The short time parameter of the NC3 sensor is mainly attributed to the co-catalysis of rGO and Pd nanoparticles. As a modified material, rGO has higher conductivity, which accelerates the transfer of charge carriers and reduces the response time and recovery time. The detection limit (DL) is an important indicator that reflects the sensing performance^36^. The lower limit of detection (DL) is defined as the minimum concentration at which the response differs significantly from the noise signal (typically DL is 3 times the noise standard deviation). Sensor noise can be calculated by changing the relative response of the sensor over the baseline^37^. Before exposure to hydrogen, take the average value of 10 consecutive data points, and calculate the root mean square deviation according to Formula (2) as 1.05 × 10^−3^

2 $${\rm{RMS}}_{{\rm{noise}}} = \sqrt {\frac{{S^2}}{N}}$$

where ${\rm{RMS}}_{{\rm{noise}}}$ is the root mean square error and $N$ is the number of data points. The detection limit is defined as Formula (3):

3 $${\rm{DL}} = 3\frac{{{\rm{RMS}}_{{\rm{noise}}}}}{{{\rm{Slope}}}}$$

According to the definition of the detection limit, for the NC3 sensor, DL = 3 × 1.05 × 10^−3^/0.067 = 0.047 ppm is calculated by Formula (3). Figure 7c shows the transient response curve of the NC3 sensor to 50 ppb H_2_ at 380 °C. The actual detection limit is basically consistent with the theoretical calculation result.

For gas sensors, selectivity is an important parameter in practical applications, as it reflects the ability of the sensor to resist gas interference and whether it can respond specifically to the target gas. Therefore, we tested the response of the NC3 sensor to 100 ppm H_2_ and other interfering gases at the same concentration (100 ppm formaldehyde, n-butane, toluene, carbon dioxide) at 380 °C. Figure 7d shows a bar graph of the sensing responses of six different sensors to five test gases. The sensor without rGO modification responds almost equally to hydrogen (4.73), formaldehyde (5.45), n-butane (2.07), toluene, and (1.98) carbon dioxide (1.43), with poor discrimination for hydrogen. The response values of the NC3 sensor to hydrogen, formaldehyde, n-butane, toluene, and carbon dioxide are 9.42, 2.74, 1.08, 1.75, and 1.67, respectively. The sensing response of the NC3 sensor to hydrogen is enhanced over that of the other sensors, and the response to other interfering gases is significantly reduced, indicating superior selectivity. Therefore, the NC3 sensor not only has a fast response speed and low detection limit but also has excellent selectivity.

**Fig. 8:**
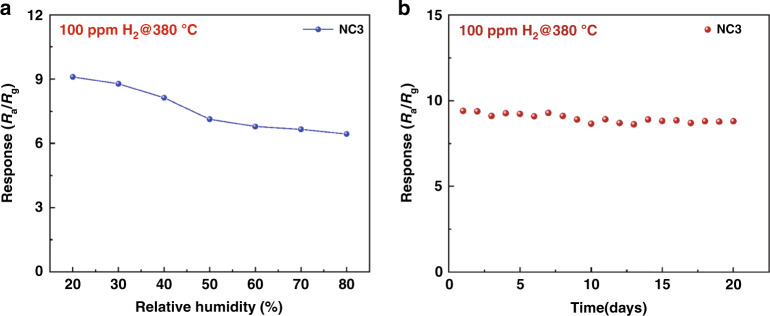
**Effect of relative humidity to response and stability of NC3 sensor. a** Response of the NC3 sensor under different relative humidities to 100 ppm H_2_ at 380 °C. **b** Stability of the NC3 sensor to 100 ppm H_2_ at 380 °C.

Figure 8a shows the response of the NC3 sensor to 100 ppm hydrogen at 380 °C under different relative humidity conditions. The response of the NC3 sensor clearly decreases with increasing relative humidity in the range of 20–80%. When the relative humidity reaches 80%, the NC3 sensor response decreases to 70.7% of the response at 20% RH. The result shows that the water molecules on the surface of the material under high relative humidity make the chemisorption of oxygen and hydrogen molecules difficult; thus, high relative humidity is not conducive to the redox reaction, resulting in decreased performance. In addition, at high relative humidity, chemisorbed oxygen ions react with water molecules on the surface of the material, reducing the baseline resistance of the sensor and resulting in a reduced sensor response. This phenomenon was observed during the experiment; as the relative humidity increased, the baseline resistance of the NC3 sensor decreased continuously.

The long-term stability of the sensor is another indispensable factor of practical application, so a study on the long-term stability of the NC3 sensor was conducted, as plotted in Fig. 8b. The response of the sensor to 100 ppm hydrogen at 380 °C was measured over 20 days. As shown by the results, the sensor was stable and exhibited only a very small decrease in response within 20 days. The maximum attenuation of the response value of the NC3 sensor was only 8.5%. The good stability of the sensor is attributed to the structural stability of the ZnO/SnO_2_ material and the co-promotion by Pd nanoparticles and rGO.

Table 2 summarizes the comparison of the hydrogen-sensing properties of different gas-sensing materials. It was observed that the proposed Pd-doped rGO/ZnO-SnO_2_ sensor exhibits outstanding hydrogen gas sensing performance. Compared with those reported in prior studies^26,38,39^, the proposed sensor shows higher response values at the same H_2_ concentration. In addition, the most critical point is that the response time and recovery time of the Pd-doped rGO/ZnO-SnO_2_ sensor proposed in this work are much shorter than those of the gas sensors in Table 2 and have an extremely low detection limit (0.05 ppm), which can achieve fast and low concentration hydrogen detection. This study provides an effective way to improve the hydrogen sensing performance of metal oxide nanomaterials, especially in terms of low time parameters and low detection limits.

**Table 2:** A comparison of the sensing performance of sensors based on the reported literature and our work (*T*: operating temperature, *C*: H_2_ concentration, Res/rec time: response/recovery time, LOD: limit of detection).

Sensing material	*T* (°C)	*C* (ppm)	Response	Res/rec time (s)	LOD (ppm)	Response formula	Ref.
SnO_2_	330	200	10.3	42.4/42.8	1	*R*_a_/*R*_g_	^26^
Ag/ZnO	250	300	4.79	175/655	5	(*R*_a_ − *R*_g_)/*R*_g_	^38^
RGO/ZnO	150	200	3.5	22/90	10	*R*_a_/*R*_g_	^39^
Pd/SnO_2_	300	250	26	5/50	25	*R*_a_/*R*_g_	^43^
SnO_2_/Pd	300	100	56	22/164	—	*R*_a_/*R*_g_	^44^
Biomorphic porous SnO_2_	240	200	17.2	6/10	—	*R*_a_/*R*_g_	^45^
ZnO–SnO_2_	330	100	10	69/—	—	*R*_a_/*R*_g_	^46^
Pd-doped rGO/ZnO-SnO_2_	380	100	9.4	4/8	0.05	*R*_a_/*R*_g_	Present work

### Gas sensing mechanism

A gas-sensing mechanism is proposed to explain the excellent performance of the Pd-doped rGO/ZnO-SnO_2_ nanocomposite sensor. The gas sensing mechanism of the resistive gas sensor is the change in resistance when the target gas exists, and the microscopic manifestation is the transfer of electrons on the surface of the material during the hydrogen adsorption and desorption process. When the composite material is exposed to air, the oxygen molecules in the air adsorb onto the surface of the material^40^. Since oxygen molecules have a strong affinity for electrons, the electrons are extracted from the conduction band of the material and converted into various oxygen ions depending on the temperature, as follows:

4 $${\rm{O}}_{2\left( {{\rm{gas}}} \right)} \to {\rm{O}}_{2\left( {{\rm{ads}}} \right)}\;{\rm{or}}\;2{\rm{O}}_{({\rm{ads}})}$$

5 $${\rm{O}}_{2({\rm{ads}})} + {\rm{e}}^ - \to {\rm{O}}_{2({\rm{ads}})}^ -$$

6 $${\rm{O}}_{({\rm{ads}})} + {\rm{e}}^ - \to {\rm{O}}_{({\rm{ads}})}^ -$$

When the composite material is exposed to hydrogen, hydrogen molecules combine with oxygen ions adsorbed on the surface of the material, releasing electrons back to the conduction band of the material, increasing the carrier concentration, reducing the width of the electron depletion layer, and reducing the resistance of the material^41^, as shown below:

7 $${\rm{H}}_{2({\rm{gas}})} \to 2{\rm{H}}_{({\rm{ads}})}$$

8 $$4{\rm{H}}_{({\rm{ads}})} + {\rm{O}}_{2({\rm{ads}})}^ - \to 2{\rm{H}}_2{\rm{O}} + {\rm{e}}^ -$$

9 $$2{\rm{H}}_{({\rm{ads}})} + {\rm{O}}_{({\rm{ads}})}^ - \to {\rm{H}}_2{\rm{O}} + {\rm{e}}^ -$$

**Fig. 9:**
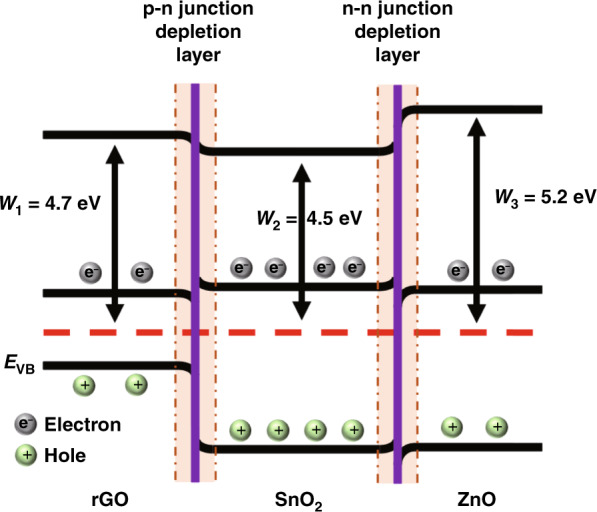
**Gas sensing mechanism of Pd-doped rGO/ZnO-SnO**_**2**_ **based sensor.** Energy band structures of the rGO/ZnO-SnO_2_ p-n-n heterojunction.

To further illustrate the sensing mechanism of the material, Fig. 9 presents the energy band diagram of the rGO/ZnO-SnO_2_ matrix material. Both ZnO and SnO_2_ are n-type semiconductors and participate in the formation of n-n heterojunctions in the composite. Since the work function of ZnO is larger than that of SnO_2_, to achieve Fermi level balance, the electrons in the conduction band of SnO_2_ transfer to the conduction band of ZnO, causing energy level bending at the junction of the two materials, forming an electron depletion layer and increasing the potential barrier, which in turn increases the substrate resistance^42^. Additionally, the modification of decorated rGO forms a p-n-n ternary heterojunction based on the n-n heterojunction, adding a p-n depletion layer and further increasing the substrate resistance^30^. In addition, the high specific surface area of rGO increases the number of active sites, which ultimately increases the adsorption capacity of hydrogen molecules. With the increase in the adsorption capacity of the hydrogen molecules, more electrons return to the conduction band of the material, which further reduces the amount of material in the target gas resistance, effectively improving the sensing response.

**Fig. 10:**
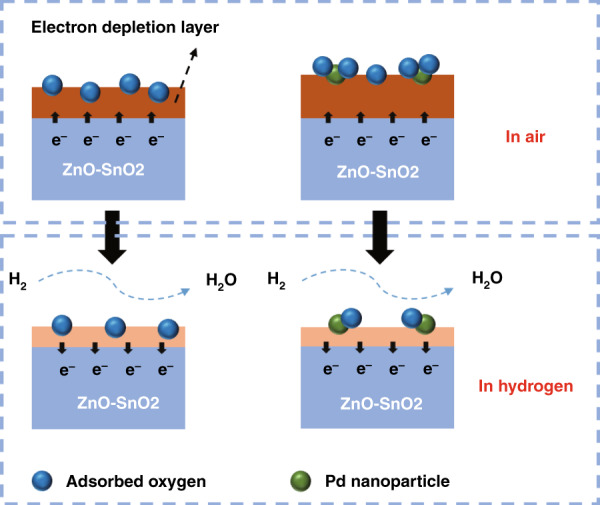
**Gas sensing mechanism of Pd-doped rGO/ZnO-SnO2 based sensor.** Gas sensing mechanism action diagram of Pd nanoparticles.

Apparently, the affinity of Pd nanoparticles to hydrogen also plays a crucial role in the highly sensitive response to hydrogen (Fig. 10). The “spillover effect” of Pd nanoparticles can adsorb more oxygen molecules and increase the initial barrier height. In addition, there is a Schottky barrier between Pd and the matrix material in the air, and the dissociated hydrogen atoms convert Pd into PdH_x_. As a result, the work function of Pd is reduced, resulting in a lower Schottky barrier height.

## Conclusion

In summary, Pd-doped rGO/ZnO-SnO_2_ nanocomposites were successfully prepared by a one-step hydrothermal method and L-ascorbic acid reduction method in our work, and the prepared sensor was applied to the gas sensing test of H_2_. The Pd-doped rGO (3 wt%)/ZnO-SnO_2_ sample achieved a response of 9.4–100 ppm H_2_ at 380 °C, with a fast linear concentration response in the range of 50–500 ppm H_2_. In addition, the sensor had extremely low time parameters (the response time and recovery time to 100 ppm H_2_ at 380 °C were 4 and 8 s, respectively) and an extremely low detection limit (50 ppb). This is the biggest highlight of this sensor. The superior gas sensing performance of this sensor is mainly attributed to the heterostructure between rGO and ZnO and SnO_2_ and the excellent electrical and physical properties of rGO. This work can lay the foundation for other researchers to explore future high-performance H_2_ sensors with ultralow time parameters and ultralow detection limits.
